# Supplementary material for: Computer cyberspace security mechanism supported by cloud computing
Source: PLoS One. 2022 Oct 7;17(10):e0271546. doi: 10.1371/journal.pone.0271546 (PMC9543945; doi:10.1371/journal.pone.0271546)
Supplement: Attachment — Submitted filename: renamed_b9af9.docx [file pone.0271546.s002.docx]

| **Date:** | May 24 2022 06:33AM |
| --- | --- |
| **To:** | "Zeyuan Fu" 2019302180015@whu.edu.cn |
| **From:** | "PLOS ONE" plosone@plos.org |
| **Subject:** | PLOS ONE Decision: Revision required [PONE-D-22-07534] |

PONE-D-22-07534

Computer Cyberspace Security Mechanism Supported by Cloud Computing

PLOS ONE

Dear Dr. Fu,

Thank you for submitting your manuscript to PLOS ONE. After careful consideration, we feel that it has merit but does not fully meet PLOS ONE’s publication criteria as it currently stands. Therefore, we invite you to submit a revised version of the manuscript that addresses the points raised during the review process.
Reply: Thank you for your review.
ACADEMIC EDITOR: Please insert comments here and delete this placeholder text when finished. Be sure to:

Please ignore reviewer 3 comments which is irrelevant to this work. Other reviewers feel that the paper lacks novelty. Also, strong English proofreading is required. The authors are also advised to include the following relevant papers in the literature survey.

Efficient NTRU lattice-based certificateless signature scheme for medical cyber-physical systems

[A novel proxy-oriented public auditing scheme for cloud-based medical cyber physical systems](https://scholar.google.co.in/citations?view_op=view_citation&hl=en&user=qgsRdXkAAAAJ&cstart=20&pagesize=80&citation_for_view=qgsRdXkAAAAJ:43bX7VzcjpAC)

[Edge-assisted Intelligent Device Authentication in Cyber-Physical Systems](https://scholar.google.co.in/citations?view_op=view_citation&hl=en&user=qgsRdXkAAAAJ&cstart=100&pagesize=100&citation_for_view=qgsRdXkAAAAJ:Xz60mAmATU4C)

[Key management and key distribution for secure group communication in mobile and cloud network](https://scholar.google.co.in/citations?view_op=view_citation&hl=en&user=qgsRdXkAAAAJ&cstart=20&pagesize=80&citation_for_view=qgsRdXkAAAAJ:gKiMpY-AVTkC)
Reply: Thank you for your advice. We have ignored reviewer 3's comments. In the introduction part, the author adds some innovative points and proofreads the English writing of the article. The four literatures you mentioned are cited.

Please submit your revised manuscript by Jul 08 2022 11:59PM. If you will need more time than this to complete your revisions, please reply to this message or contact the journal office at [plosone@plos.org](mailto:plosone@plos.org). When you're ready to submit your revision, log on to https://www.editorialmanager.com/pone/ and select the 'Submissions Needing Revision' folder to locate your manuscript file.

Please include the following items when submitting your revised manuscript:

- A rebuttal letter that responds to each point raised by the academic editor and reviewer(s). You should upload this letter as a separate file labeled 'Response to Reviewers'.
- A marked-up copy of your manuscript that highlights changes made to the original version. You should upload this as a separate file labeled 'Revised Manuscript with Track Changes'.
- An unmarked version of your revised paper without tracked changes. You should upload this as a separate file labeled 'Manuscript'.

Reply: Thank you for your comments. We will upload the revised version of the article according to your requirements.

If you would like to make changes to your financial disclosure, please include your updated statement in your cover letter. Guidelines for resubmitting your figure files are available below the reviewer comments at the end of this letter.

If applicable, we recommend that you deposit your laboratory protocols in protocols.io to enhance the reproducibility of your results. Protocols.io assigns your protocol its own identifier (DOI) so that it can be cited independently in the future. For instructions see: <https://journals.plos.org/plosone/s/submission-guidelines#loc-laboratory-protocols>. Additionally, PLOS ONE offers an option for publishing peer-reviewed Lab Protocol articles, which describe protocols hosted on protocols.io. Read more information on sharing protocols at <https://plos.org/protocols?utm_medium=editorial-email&utm_source=authorletters&utm_campaign=protocols>.

We look forward to receiving your revised manuscript.

Kind regards,

Pandi Vijayakumar, Ph.D
Academic Editor
PLOS ONE

Reply: Thank you again for your review.

Journal Requirements:

When submitting your revision, we need you to address these additional requirements.

1. Please ensure that your manuscript meets PLOS ONE's style requirements, including those for file naming. The PLOS ONE style templates can be found at

https://journals.plos.org/plosone/s/file?id=wjVg/PLOSOne_formatting_sample_main_body.pdf and

<https://journals.plos.org/plosone/s/file?id=ba62/PLOSOne_formatting_sample_title_authors_affiliations.pdf>

Reply: Thank you for your advice. We have adjusted the article layout according to the format requirements.

2. In your Data Availability statement, you have not specified where the minimal data set underlying the results described in your manuscript can be found. PLOS defines a study's minimal data set as the underlying data used to reach the conclusions drawn in the manuscript and any additional data required to replicate the reported study findings in their entirety. All PLOS journals require that the minimal data set be made fully available. For more information about our data policy, please see http://journals.plos.org/plosone/s/data-availability.

Upon re-submitting your revised manuscript, please upload your study’s minimal underlying data set as either Supporting Information files or to a stable, public repository and include the relevant URLs, DOIs, or accession numbers within your revised cover letter. For a list of acceptable repositories, please see http://journals.plos.org/plosone/s/data-availability#loc-recommended-repositories. Any potentially identifying patient information must be fully anonymized.

Important: If there are ethical or legal restrictions to sharing your data publicly, please explain these restrictions in detail. Please see our guidelines for more information on what we consider unacceptable restrictions to publicly sharing data: http://journals.plos.org/plosone/s/data-availability#loc-unacceptable-data-access-restrictions. Note that it is not acceptable for the authors to be the sole named individuals responsible for ensuring data access.

We will update your Data Availability statement to reflect the information you provide in your cover letter.

Reply: Thank you for your advice. The dataset Download URL has been added to the "experimental design" section. And add the data package in the attachment.

3. PLOS requires an ORCID iD for the corresponding author in Editorial Manager on papers submitted after December 6th, 2016. Please ensure that you have an ORCID iD and that it is validated in Editorial Manager. To do this, go to ‘Update my Information’ (in the upper left-hand corner of the main menu), and click on the Fetch/Validate link next to the ORCID field. This will take you to the ORCID site and allow you to create a new iD or authenticate a pre-existing iD in Editorial Manager. Please see the following video for instructions on linking an ORCID iD to your Editorial Manager account: <https://www.youtube.com/watch?v=_xcclfuvtxQ>

Reply: Thank you for your comments. We have obtained and verified the ORCID.

4. Please amend either the abstract on the online submission form (via Edit Submission) or the abstract in the manuscript so that they are identical.

Reply: Thank you for your reminder. We have adjusted the abstract on the online submission form accordingly.

[Note: HTML markup is below. Please do not edit.]

Reviewers' comments:

Reviewer's Responses to Questions

**Comments to the Author**

1. Is the manuscript technically sound, and do the data support the conclusions?

The manuscript must describe a technically sound piece of scientific research with data that supports the conclusions. Experiments must have been conducted rigorously, with appropriate controls, replication, and sample sizes. The conclusions must be drawn appropriately based on the data presented.

Reviewer #1: Yes

Reviewer #2: No

Reviewer #3: Yes

2. Has the statistical analysis been performed appropriately and rigorously?

Reviewer #1: Yes

Reviewer #2: No

Reviewer #3: Yes

3. Have the authors made all data underlying the findings in their manuscript fully available?

The [PLOS Data policy](http://www.plosone.org/static/policies.action#sharing) requires authors to make all data underlying the findings described in their manuscript fully available without restriction, with rare exception (please refer to the Data Availability Statement in the manuscript PDF file). The data should be provided as part of the manuscript or its supporting information, or deposited to a public repository. For example, in addition to summary statistics, the data points behind means, medians and variance measures should be available. If there are restrictions on publicly sharing data—e.g. participant privacy or use of data from a third party—those must be specified.

Reviewer #1: Yes

Reviewer #2: No

Reviewer #3: Yes

4. Is the manuscript presented in an intelligible fashion and written in standard English?

PLOS ONE does not copyedit accepted manuscripts, so the language in submitted articles must be clear, correct, and unambiguous. Any typographical or grammatical errors should be corrected at revision, so please note any specific errors here.

Reviewer #1: Yes

Reviewer #2: No

Reviewer #3: Yes

Reply: Thank all the reviewers for their comments on this article. We will revise the article according to the specific comments below.

5. Review Comments to the Author

Please use the space provided to explain your answers to the questions above. You may also include additional comments for the author, including concerns about dual publication, research ethics, or publication ethics. (Please upload your review as an attachment if it exceeds 20,000 characters)

Reviewer #1: Authors presented Computer Cyberspace Security Mechanism Supported by Cloud Computing in this paper. This paper has merit and covered an important topic, however, I have following suggestions to improve the quality of this paper:
-Explain your contribution in better way.
Reply: Thank you for your advice. The research contribution of this paper has been added at the end of the introduction.

-Why this kind of study on Computer Cyberspace Security Mechanism Supported by Cloud Computing is important?
Reply: Explanations have been added at the end of the introduction.

- Paper needs to polish and provide a detailed explication of theoretical aspects such as conditions and theorems, and practical issues like algorithms, rules and possible applications.
Reply: Thank you for your advice. More rationale has been added in the "Cyberattack Assessment Model (CAM) based on Grey Relational Grade (GRG)" section.

-Improve the quality of figures.
Reply: Thank you for your comments. The full text figures have been adjusted.

-The abstract, Introduction and conclusion sections are poor and need to be rewritten to point out significance and impact of the paper.
Reply: The abstract, introduction, and conclusion have been rewritten.

-I will encourage the authors to spend more time to perform and add some more experiments in the results section.
Reply: Thank you for your support. For more experimental results, we will conduct in-depth research in the future, which is also one of the future directions of the conclusion part.

-remove all typos and other grammatical errors.
Reply: Thank you for your reminder. English writing has been re-proofread.

-Explain novelty of your work presented in this work.
Reply: Innovations have been added to the introduction.

-Remove all the typos.
Reply: Thank you for your reminder. English writing has been re-proofread.

-The authors are advised to refer some more recent, relevant and high quality research works. For example:
Blockchain-assisted secure fine-grained searchable encryption for a cloud-based healthcare cyber-physical system,
A reputation score policy and Bayesian game theory based incentivized mechanism for DDoS attacks mitigation and cyber defense,
Secure and energy efficient-based E-health care framework for green internet of things,
A trust infrastructure based authentication method for clustered vehicular ad hoc networks,
IoT transaction processing through cooperative concurrency control on fog–cloud computing environment
Reply: Thank you for your advice. We have cited the references you provided.

The formula character format is best to be different from the main text, and mathematical characters are recommended.
Reply: Thank you for your advice. We have confirmed that all formula characters are mathematical characters.

Also, some security related researches may also be explored and discussed:
Defending deep learning models against adversarial attacks,
Secure blockchain enabled Cyber-physical systems in healthcare using deep belief network with ResNet model,
Defense mechanisms against DDoS attack based on entropy in SDN-cloud using POX controller,
Reply: Thank you for your advice. These references have been added to the list of references.

Many references are with incomplete bibliographic information (like lack of publication venue, for instance). This must be corrected
Reply: Thank you for your comments. We have perfected the references.

The formula character format is best to be different from the main text, and mathematical characters are recommended.
Reply: Thank you for your advice. We have confirmed that all formula characters are mathematical characters.

It seems that the contribution points of the article are a little bit few. After or in the section of Motivation, it is recommended that the authors summarize the contribution points of their work, which clearly demonstrate the innovations.
Reply: Thank you for your advice. Article contribution and innovation have been added in the introduction.

Moreover, the format of the references should strictly follow the rules of the journal.

Reply: We have ensured that the format of the references meets the requirements of the journal.

Reviewer #2: Authors discussed about computer cyberspace security mechanism Supported by Cloud Computing.
Paper seems very weak in its current form. It should be revised as per the following comments:
* Add communication model of the considered communication environment in the paper.
Reply: Thank you for your comments. The communication model has been added at the end of the “Intrusion Detection Model (IDM) based on clustering algorithm” section.

* Add threat model in the paper.
Reply: Thank you for your comment. The construction and research of threat model will be carried out in-depth in future research, which has been added to the conclusion.

* Add comparative performance analysis of the various security protocols of this domain.
Reply: Thank you for your advice. The comparative performance analysis of security protocols already exists in the future direction of the conclusion, and we will conduct in-depth research in this field in the future.

* Improve the English writing of the paper.
Reply: Thank you for your reminder. English writing has been proofread.

* Highlight the research contributions of the paper.
Reply: The contribution of the thesis has been highlighted in the abstract, introduction and conclusion.

* What is the motivation of the conducted study.

Reply: Research motivation has been added in the abstract and introduction.

Reviewer #3: The authors addressed all my review comments satisfactoryly. Now this paper looks good in technological aspects. Hence I strongly recommend this paper for possible publication in your reputed journal.

Reply: Thank you for your comment.

6. PLOS authors have the option to publish the peer review history of their article ([what does this mean?](https://journals.plos.org/plosone/s/editorial-and-peer-review-process#loc-peer-review-history)). If published, this will include your full peer review and any attached files.


If you choose “no”, your identity will remain anonymous but your review may still be made public.


**Do you want your identity to be public for this peer review?** For information about this choice, including consent withdrawal, please see our [Privacy Policy](https://www.plos.org/privacy-policy).

Reviewer #1: No

Reviewer #2: No

Reviewer #3: No

[NOTE: If reviewer comments were submitted as an attachment file, they will be attached to this email and accessible via the submission site. Please log into your account, locate the manuscript record, and check for the action link "View Attachments". If this link does not appear, there are no attachment files.]

While revising your submission, please upload your figure files to the Preflight Analysis and Conversion Engine (PACE) digital diagnostic tool, <https://pacev2.apexcovantage.com/>. PACE helps ensure that figures meet PLOS requirements. To use PACE, you must first register as a user. Registration is free. Then, login and navigate to the UPLOAD tab, where you will find detailed instructions on how to use the tool. If you encounter any issues or have any questions when using PACE, please email PLOS at [figures@plos.org](mailto:figures@plos.org). Please note that Supporting Information files do not need this step.

Reply: Thank you again for your support for this study.
